# Multi-dimensional sleep patterns predict complications and mortality in chronic kidney disease patients: a prospective study from the UK Biobank

**DOI:** 10.3389/fpubh.2026.1889689

**Published:** 2026-08-18

**Authors:** Kaixin Lei, Jiameng Li, Lifan Xu, Yi Teng, Chenxi Lu, Chunxia Qiao, Wen Guo

**Affiliations:** 1 The Center of Gerontology and Geriatrics, Sichuan University West China Hospital, Chengdu, Sichuan, China; 2 West China School of Medicine, Sichuan University West China Hospital, Chengdu, Sichuan, China; 3 National Center for Chronic Non-communicable Disease Control and Prevention, Chinese Center for Disease Control and Prevention, Beijing, China; 4 Mental Health Center and Psychiatric Laboratory, Sichuan University West China Hospital, Chengdu, Sichuan, China

**Keywords:** chronic kidney disease, latent class analysis, mortality, outcomes, sleep

## Abstract

**Background:**

The complex interplay of multiple sleep factors may affect the risk of complications and mortality in the chronic kidney disease (CKD) population. However, few studies have examined multi-dimensional sleep health in CKD populations.

**Methods:**

Our cohort study included 15,809 participants with laboratory-observed kidney dysfunction or a CKD diagnosis during 2006–2010 from the UK Biobank. Incidence of adverse outcomes was identified via the International Classification of Diseases, 10th Edition (ICD-10) till 2022. The multi-dimensional sleep health was evaluated through an *a priori* sleep health score (SHS) and sleep health cluster categorized by latent class analysis. The relationships between multi-dimensional sleep health, each sleep factor, and outcomes in CKD were estimated through Cox proportional hazards models.

**Results:**

A total of 2,416 deaths, 2,343 anemia, 4,087 CVD, 940 cognitive impairment, and 314 hyperparathyroidism occurred during the follow-up. After adjusting for potential confounders, higher SHS was significantly associated with decreased risks of anemia, CVD, and hyperparathyroidism (all *p* < 0.05). Severe insomnia with short or long sleep duration and severe disturbed sleep with multiple dysfunctions were significantly associated with 19–54% higher risks of anemia, CVD and hyperparathyroidism. Each sleep factor indicated great heterogeneity in association with adverse outcomes in the CKD population, while gender and kidney function interact with some of these exposures.

**Conclusion:**

Poor multi-dimensional sleep health, either estimated by SHS or characterized by sleep clusters, was associated with poor prognosis in the CKD population. The specific sleep profiles in the CKD population contribute to pinpointing high-risk subgroups and providing targeted interventions.

## 1 Introduction

Chronic kidney disease (CKD), identified by estimated glomerular filtration rate (eGFR) < 60 mL/min/1.73 m^2^ or pathological evidence of kidney damage with relatively preserved kidney function for longer than 3 months, has emerged as a worldwide health issue with a global prevalence of 10–16% (1, 2). The life expectancy loss caused by CKD has reached the 17th and kept on escalating over the past decades, warranting attention for public healthcare (3). Typically, studies have demonstrated elevated risk profiles for multiple organ dysfunctions in the CKD population, including cardiovascular disease (CVD) (4), anemia (5), cognitive impairment (6) and hyperparathyroidism (7), which, to a large extent, contribute to the poor prognosis of this population.

Additionally, sleep-related problems such as insomnia or abnormal sleep duration are quite prevalent among CKD patients (8, 9). Emerging evidence has proved that sleep disorders could further compound the burden of high morbidity and mortality, including cognitive dysfunction (10), health-related quality of life (11), rapid decline of kidney function (12), as well as increased CVD incidence (13). However, these studies inadequately investigated the relationships between sleep disorders and CKD-related outcomes as they exclusively considered the individual impact of each sleep factor, overlooking the coexistence of different sleep phenotypes. Additionally, the relationships between sleep health and other CKD-related outcomes, including anemia, hyperparathyroidism, or mortality, have not been fully elucidated. As a consequence, it is essential to establish a holistic methodology for a full understanding of sleep dysfunction and health-related outcomes in the CKD population.

The multi-dimensional sleep health framework is launched for evaluation of interactions between different sleep health factors, as they rarely occur in isolation (14). Conversely, several sleep factors may cluster together to facilitate a distinct sleep pattern with significant clinical implications (15). For instance, insomnia, a common sleep disorder in CKD, is highly heterogeneous (8, 16). The comorbidity of insomnia and sleep apnea further aggravated risks for diabetes and CVD than either one, presenting challenges for management of both disorders (16). Beyond these, short sleep duration in combination with insomnia was regarded as a biologically more severe phenotype correlated with poor cardiometabolic and neurocognitive outcomes (17, 18). Consequently, exclusive consideration of individual sleep dimensions would inadequately capture the etiological complexities and shadow the strong impacts of sleep health in CKD patients, leading to potentially misleading conclusions.

To bridge this research gap, we used national population data from the UK Biobank to derive sleep health phenotypes within the multi-dimensional sleep framework to explore the differences between sleep phenotypes and health-related outcomes in CKD. Furthermore, we performed eGFR and sex stratification to observe how different sleep patterns or factors may interact with residual kidney function and sex in CKD outcomes.

## 2 Methods

### 2.1 Study population

The UK Biobank is a large national cohort launched in 2006 with registration of over 500,000 residents aged 40–69 years in the UK. This study provides comprehensive data on demographic, experimental laboratories, physical, imaging, and genetic data. At baseline (2006–2010), the participants filled in the comprehensive questionnaires (where sleep health information was collected), underwent physical examinations, and offered biological samples for subsequent experimental assays and genetic detections. Further details of this dataset were published in the previous article (19).

The inclusion criteria of our study are: (1) participants with CKD. CKD was defined as either eGFR < 60 mL/min/1.73 m^2^ (calculated by the CKD-EPI equation 2021) (20), urinary albumin creatinine ratio (UACR) > 30 mg/g, clinical diagnosis based on International Classification of Diseases, 10th Edition (ICD-10), or Office of Population Censuses and Surveys Classification of Interventions and Procedures-version 4 (Supplementary Table S1). (2) patients participate in an in-touch screen sleep questionnaire evaluation. (3) participants aged 40–69 years at baseline. These participants were excluded from final analysis: (1) outcomes occurred prior to baseline. (2) patients who did not answer all sleep factor questions.

This study is approved by the Declaration of Helsinki. Any analysis based on the UK Biobank cohort was permitted by the ethical approval from the NHS National Research Ethics Service on 17 June 2011 (Ref 11/NW/0382) and its extension on 18 June 2021 (Ref 21/NW/0157). All participants offered their written informed consent.

### 2.2 Sleep assessment

A touch-screen questionnaire was sent to participants with evaluation on sleep health in seven vital aspects: (1) sleep period, (2) insomnia symptoms, (3) non-restorative sleep rhythm, (4) snoring, (5) sleepiness during daytime, (6) napping, and (7) chronotype. The details of the evaluation questions are shown in Supplementary Table S2. According to the statistical analysis guidance, we excluded answers with sleep durations longer than 14 h or shorter than 3 h per day. Sleep duration was categorized as medium (7–8 h sleep) and abnormal sleep duration (<7 h or >8 h). To reflect sleep chronotype, we identified intermediate chronotype and abnormal chronotype (including “evening” or “morning” chronotypes). The other five factors were also identified in binary ways. As sleep disorders more frequently occur in older people, older participants who reported “sometimes” experiences of sleepiness, napping, and insomnia were considered as “rarely or never.”

Sleep health score (SHS) was defined *a priori* with 1 point for satisfying the following items and 0 otherwise, which simplified clinical generalization: 7–8 h of sleep duration, absence of insomnia symptoms, sleepiness, or napping, no self-reported snoring, restorative sleep, and an intermediate chronotype (17). Lower SHS represented poorer sleep health. Following these criteria, we categorized sleep health into four classifications: healthiest (6-7), mild (5), moderate (3-4), and severe (0–2).

### 2.3 Outcome ascertainment

The targeted outcomes in the present study included incident complications in CKD, all-cause mortality, and CVD mortality. Incident complications included anemia, CVDs, hyperparathyroidism and cognitive impairment. CVD was identified as a combination of the following diseases: chronic ischemic heart disease, angina, heart attack, heart failure, atrial fibrillation, peripheral arterial disease and stroke. Similarly, cognitive impairment was determined as mild cognitive impairment and dementia (including Alzheimer’s disease, vascular dementia, frontotemporal dementia, and dementia of other causes). All these diseases were obtained from the International Classification of Diseases 10 (ICD-10), with details shown in Supplementary Table S3.

Mortality outcomes were recorded as any death for any reason or CVD during the following period, and details were described in a previous report (19). In the UK Biobank, the end time of follow-up was either the occurrence of an outcome of interest or their last record, including death, loss to follow-up, and the conclusion date of this study (31 December 2022).

### 2.4 Statistical analysis

For the distribution of SHS and sleep health factors in different age groups, we utilized line plots for each sleep factor and SHS score, respectively. Subsequently, we performed latent class analysis (LCA) to consider co-effects of multiple sleep disorders with clusters of participants sharing similar sleep patterns via the R package *poLCA* (21, 22). LCA is a data-driven approach for the discovery of potential groups based on estimated membership probabilities and observed responses via the expectation–maximization algorithm (23). The large sample size in the UK Biobank cohort guaranteed that we could recognize optimal numbers of clusters and avoid overfitting. Additionally, we used spider plots to visualize characteristics of different sleep patterns.

Multivariate Cox proportional hazards models were used to estimate hazard ratios (HRs) and their 95% confidence intervals (CIs) between sleep health and targeted outcomes. In order to alleviate potential impact from confounding factors, we considered age, sex, race, hypertension, diabetes, body mass index (BMI), Townsend deprivation index, smoking, drinking and history of taking RAASi agents (Supplementary methods). These analyses were conducted to investigate the relationships between the sleep patterns from LCA analysis and sleep factors and the prognostic outcomes in CKD patients. Linear trends were performed by considering SHS as a continuous variable. Sensitivity analysis based on multivariate Cox proportional models. Gender (female or male) and eGFR (>90, 60–89, 45–59, and <45) were regarded as subgroup factors to explore whether sex and residual kidney function could impact the influences from sleep patterns and individual sleep factors. Statistical significance for subgroup interactions was defined through a likelihood ratio test comparing models with and without the multiplicative interaction term between sleep health and subgroup factors. For sensitivity analysis, we first excluded outcomes that occurred during the first 3 years of follow-up to avoid reverse causation. Furthermore, we included anxiety and depression as covariates to eliminate confounding effects from psychological factors in the CKD population. Anxiety and depression were defined via ICD-10 diagnosis and self-reports (Supplementary methods). The significance threshold for the present study was *p* < 0.05, and all statistical analyses were performed in RStudio (version 4.4.0) on the UK Biobank Research Analysis Platform.

## 3 Results

### 3.1 Baseline characteristics

**Figure 1:**
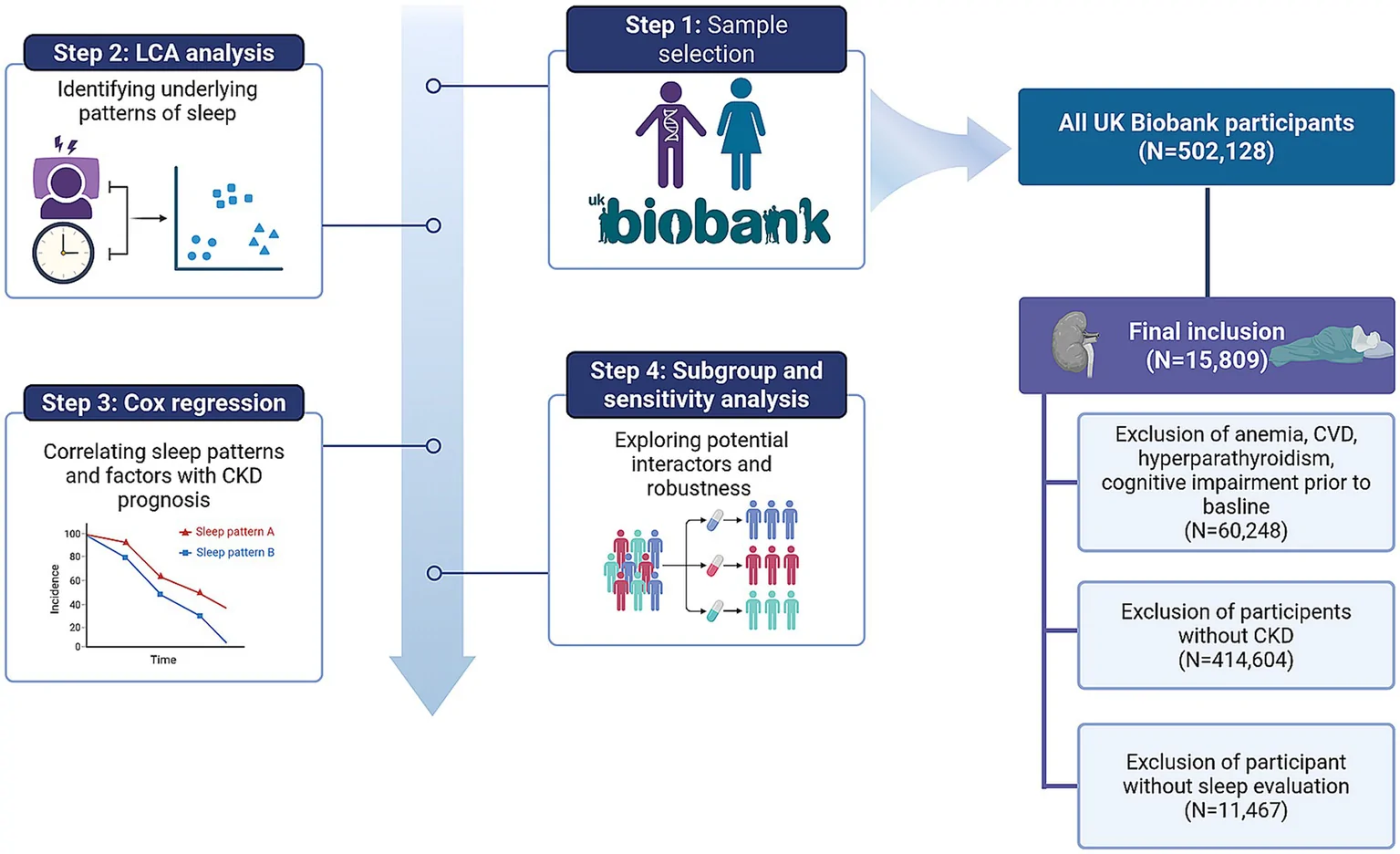
Design and sample inclusion flowchart of this study. Created in BioRender. Lei, K. (2026) https://BioRender.com/gk2e843.

As shown in Figure 1, after exclusion of 414,606 healthy participants, 60,248 outcome events occurred prior to baseline, and 11,467 participants were without data for sleep evaluation; a total of 15,809 patients were eligible for the present analysis. The average age of all participants was 61 [interquartile range (IQR), 54–66], with 92.8% identified as white (Table 1). Generally, male patients with exposure to smoking, alcohol, and use of RAASi were prone to poorer sleep (Table 1). Additionally, patients with poorer sleep quality had higher BMI, Townsend deprivation index, greater prevalence of hypertension, diabetes, depression, and anxiety.

**Table 1:** Baseline characteristics of included participants.

Demographic factors	Overall	Multi-dimensional sleep health score				*p*-value
		Healthiest	Mild	Moderate	Severe	
Number	15,809	9,269	3,919	2,412	209	
Age	61.00 [54.00, 66.00]	61.00 [54.00, 66.00]	61.00 [55.00, 66.00]	61.00 [54.00, 65.00]	60.00 [53.00, 65.00]	0.008
Sex						
Female	8,815 (55.8)	5,395 (58.2)	2084 (53.2)	1,239 (51.4)	97 (46.4)	<0.001
Male	6,994 (44.2)	3,874 (41.8)	1835 (46.8)	1,173 (48.6)	112 (53.6)	
Ethnicity						
Other	437 (2.8)	244 (2.6)	112 (2.9)	69 (2.9)	12 (5.7)	0.020
White	14,669 (92.8)	8,633 (93.1)	3,615 (92.2)	2,237 (92.7)	184 (88.0)	
Black	158 (1.0)	83 (0.9)	50 (1.3)	23 (1.0)	2 (1.0)	
Asian	454 (2.9)	264 (2.8)	120 (3.1)	63 (2.6)	7 (3.3)	
Mixed	91 (0.6)	45 (0.5)	22 (0.6)	20 (0.8)	4 (1.9)	
Creatinine	78.90 [64.30, 95.90]	78.60 [64.00, 96.20]	79.40 [65.10, 95.90]	79.40 [64.20, 95.35]	76.75 [63.32, 95.98]	0.601
eGFR	82.57 [59.07, 96.37]	82.09 [58.99, 96.00]	82.35 [59.08, 96.46]	83.97 [59.49, 97.31]	87.60 [59.46, 99.64]	0.021
Body mass index	27.90 [24.88, 31.54]	27.26 [24.40, 30.65]	28.59 [25.44, 32.21]	29.41 [25.94, 33.34]	31.35 [27.14, 35.55]	<0.001
Townsend deprivation index	−2.04 [−3.54, 0.71]	−2.19 [−3.60, 0.40]	−1.97 [−3.50, 0.92]	−1.62 [−3.42, 1.42]	−0.54 [−2.72, 2.62]	<0.001
Smoking status						
Never	8,279 (52.4)	5,195 (56.0)	1909 (48.7)	1,087 (45.1)	88 (42.1)	<0.001
Previous	5,913 (37.4)	3,294 (35.5)	1,574 (40.2)	958 (39.7)	87 (41.6)	
Current	1,617 (10.2)	780 (8.4)	436 (11.1)	367 (15.2)	34 (16.3)	
Alcohol status						
Never	961 (6.1)	579 (6.2)	220 (5.6)	141 (5.8)	21 (10.0)	<0.001
Previous	682 (4.3)	346 (3.7)	193 (4.9)	122 (5.1)	21 (10.0)	
Current	14,166 (89.6)	8,344 (90.0)	3,506 (89.5)	2,149 (89.1)	167 (79.9)	
Hypertension status						
No	8,502 (53.8)	5,323 (57.4)	1964 (50.1)	1,128 (46.8)	87 (41.6)	<0.001
Yes	7,307 (46.2)	3,946 (42.6)	1955 (49.9)	1,284 (53.2)	122 (58.4)	
Diabetes status						
No	13,930 (88.1)	8,383 (90.4)	3,415 (87.1)	1980 (82.1)	152 (72.7)	<0.001
Yes	1879 (11.9)	886 (9.6)	504 (12.9)	432 (17.9)	57 (27.3)	
RAASi use						
No	11,655 (73.7)	7,046 (76.0)	2,798 (71.4)	1,673 (69.4)	138 (66.0)	<0.001
Yes	4,154 (26.3)	2,223 (24.0)	1,121 (28.6)	739 (30.6)	71 (34.0)	
Anxiety status						
No	15,237 (96.4)	8,992 (97.0)	3,757 (95.9)	2,293 (95.1)	195 (93.3)	<0.001
Yes	572 (3.6)	277 (3.0)	162 (4.1)	119 (4.9)	14 (6.7)	
Depression status						
No	14,602 (92.4)	8,756 (94.5)	3,581 (91.4)	2,103 (87.2)	162 (77.5)	<0.001
Yes	1,207 (7.6)	513 (5.5)	338 (8.6)	309 (12.8)	47 (22.5)	
Sleep duration score						
Long or short sleep	5,528 (35.0)	1,301 (14.0)	2,283 (58.3)	1756 (72.8)	188 (90.0)	<0.001
Medium sleep	10,281 (65.0)	7,968 (86.0)	1,636 (41.7)	656 (27.2)	21 (10.0)	
Insomnia score						
Insomnia symptoms	4,733 (29.9)	981 (10.6)	1926 (49.1)	1,641 (68.0)	185 (88.5)	<0.001
Absence of insomnia	11,076 (70.1)	8,288 (89.4)	1993 (50.9)	771 (32.0)	24 (11.5)	
Non-restorative score						
Non-restorative sleep	1939 (12.3)	201 (2.2)	514 (13.1)	1,049 (43.5)	175 (83.7)	<0.001
Restorative sleep	13,870 (87.7)	9,068 (97.8)	3,405 (86.9)	1,363 (56.5)	34 (16.3)	
Sleepiness score						
Frequent daytime sleepiness	587 (3.7)	33 (0.4)	123 (3.1)	327 (13.6)	104 (49.8)	<0.001
Seldom daytime sleepiness	15,222 (96.3)	9,236 (99.6)	3,796 (96.9)	2085 (86.4)	105 (50.2)	
Snoring score						
Snoring	6,369 (40.3)	2,487 (26.8)	2085 (53.2)	1,622 (67.2)	175 (83.7)	<0.001
Non-snoring	9,440 (59.7)	6,782 (73.2)	1834 (46.8)	790 (32.8)	34 (16.3)	
Napping score						
Frequent napping	1,118 (7.1)	146 (1.6)	336 (8.6)	520 (21.6)	116 (55.5)	<0.001
Rare napping	14,691 (92.9)	9,123 (98.4)	3,583 (91.4)	1892 (78.4)	93 (44.5)	
Chronotype score						
Intermediate chronotype	1923 (12.2)	255 (2.8)	571 (14.6)	951 (39.4)	146 (69.9)	<0.001
Morning or evening chronotype	13,886 (87.8)	9,014 (97.2)	3,348 (85.4)	1,461 (60.6)	63 (30.1)	

eGFR, estimated glomerular filtration rate; RAASi, renin–angiotensin–aldosterone inhibitors.

### 3.2 Trends of multi-dimensional sleep health stratified by age and sex

The trends of multi-dimensional sleep quality are shown in Supplementary Figure S1. We observed increased morning chronotype and decreased evening chronotype, together with similar trends of non-restorative symptoms (Supplementary Figures S1A,D). Conversely, napping symptoms and sleepiness surged in older patients, with males suffering more severe symptoms than females (Supplementary Figures S1C,G). The prevalence of insomnia and short sleep duration varied across different age groups, with significant sex-related differences (Supplementary Figures S1B,E). Snoring symptoms showed an increasing trend among 40–59 years and then decreased from 59 to 75, with better performance in females (Supplementary Figure S1G). Overall, the mean SHS exhibited prevalent mild sleep dysfunction across the CKD population and was slightly higher in women than men, while sex differences initially narrowed and subsequently enlarged with age (Supplementary Figure S1H).

### 3.3 Multiple sleep health and complications in the CKD population

During the whole follow-up period, a total of 2,416 deaths, 2,343 anemia, 940 cognitive impairment, 4,087 CVD, 362 deaths due to CVD, and 314 hyperparathyroidism occurred. As shown in Table 2, SHS was significantly correlated with anemia (HR, 0.935, 95%CI, 0.904–0.967, *p* < 0.001), CVD (HR, 0.939, 95%CI, 0.915–0.964, *p* < 0.001), and hyperparathyroidism (HR, 0.904, 95%CI, 0.824–0.992, *p* = 0.033). Compared with the healthiest sleep group, mild sleep dysfunction was significantly correlated with higher all-cause mortality, anemia, and CVD (Supplementary Table S4). Moderate sleep dysfunction measured by SHS was associated with elevated risks of anemia, CVD, and hyperparathyroidism (Supplementary Table S4). Moreover, severe sleep dysfunction only correlated with higher anemia risk, further underscoring the complex impacts of multiple sleep disorders on complications in CKD.

**Table 2:** Association between SHS and health outcomes in CKD.

Outcome	Events	HR (95%CI)^a^	*p*-value	Follow-up (median, years)
All-cause mortality	2,416	0.980 (0.947–1.014)	0.235	13.46
CVD mortality	362	0.971 (0.890–1.059)	0.504	13.58
Anemia	2,343	0.935 (0.904–0.967)	<0.001	13.48
Cognitive impairment	940	0.996 (0.941–1.053)	0.875	13.62
CVD	4,087	0.939 (0.915–0.964)	<0.001	13.24
Hyperparathyroidism	314	0.904 (0.824–0.992)	0.033	13.67

The model used to investigate the association of SHS and complications in CKD was fully adjusted for age, sex, race, diabetes, hypertension, BMI, Townsend’s deprivation index, smoking, drinking, and history of taking RASSi agents.

### 3.4 Multi-dimensional sleep patterns and complications in the CKD population

**Figure 2:**
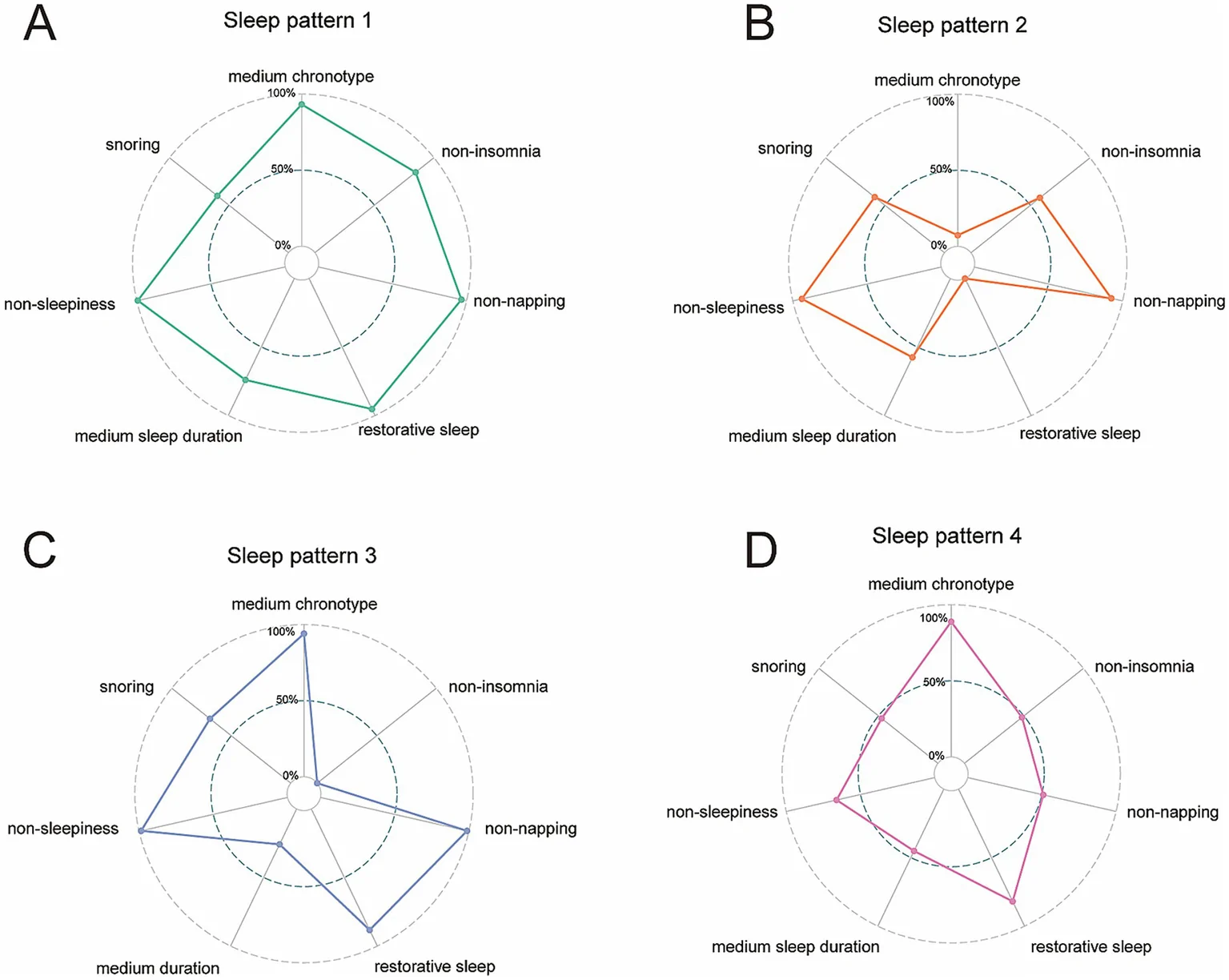
Four radar charts describing **(A)** sleep pattern 1, **(B)** sleep pattern 2, **(C)** sleep pattern 3 and **(D)** sleep pattern 4 via latent class analysis based on comparisons of sleep factors including medium chronotype, non-insomnia, non-napping, restorative sleep, medium sleep duration, non-sleepiness, and snoring.

The detailed information on LCA model selection is shown in Supplementary Table S5. Based on multiple evaluations of Bayesian Information Criterion (BIC), Akaike Information Criterion (AIC), likelihood ratio chi-square (Gsq), and log-likelihood (Loglik) tests, LCA analysis identified four clusters that had reliable abilities for differentiation of sleep patterns (BIC = 94,155; AIC = 93,917; Gsq = 251; logLik = −46,928). Under the LCA analysis framework, we identified four multi-dimensional sleep patterns as follows (Figure 2): (1) sleep pattern 1 (*N* = 11,896): relative healthy sleep paradigm; (2) sleep pattern 2 (*N* = 952): morning or evening chronotype (92.7%), non-restorative sleep (99.9%), and insomnia (42.1%); (3) sleep pattern 3 (*N* = 2016): insomnia (99.9%) accompanying with short or long sleep duration (74.1%); and (4) sleep pattern 4 (*N* = 945): severe disturbed sleep with multiple impairment and multi-dimensional dysfunction.

**Figure 3:**
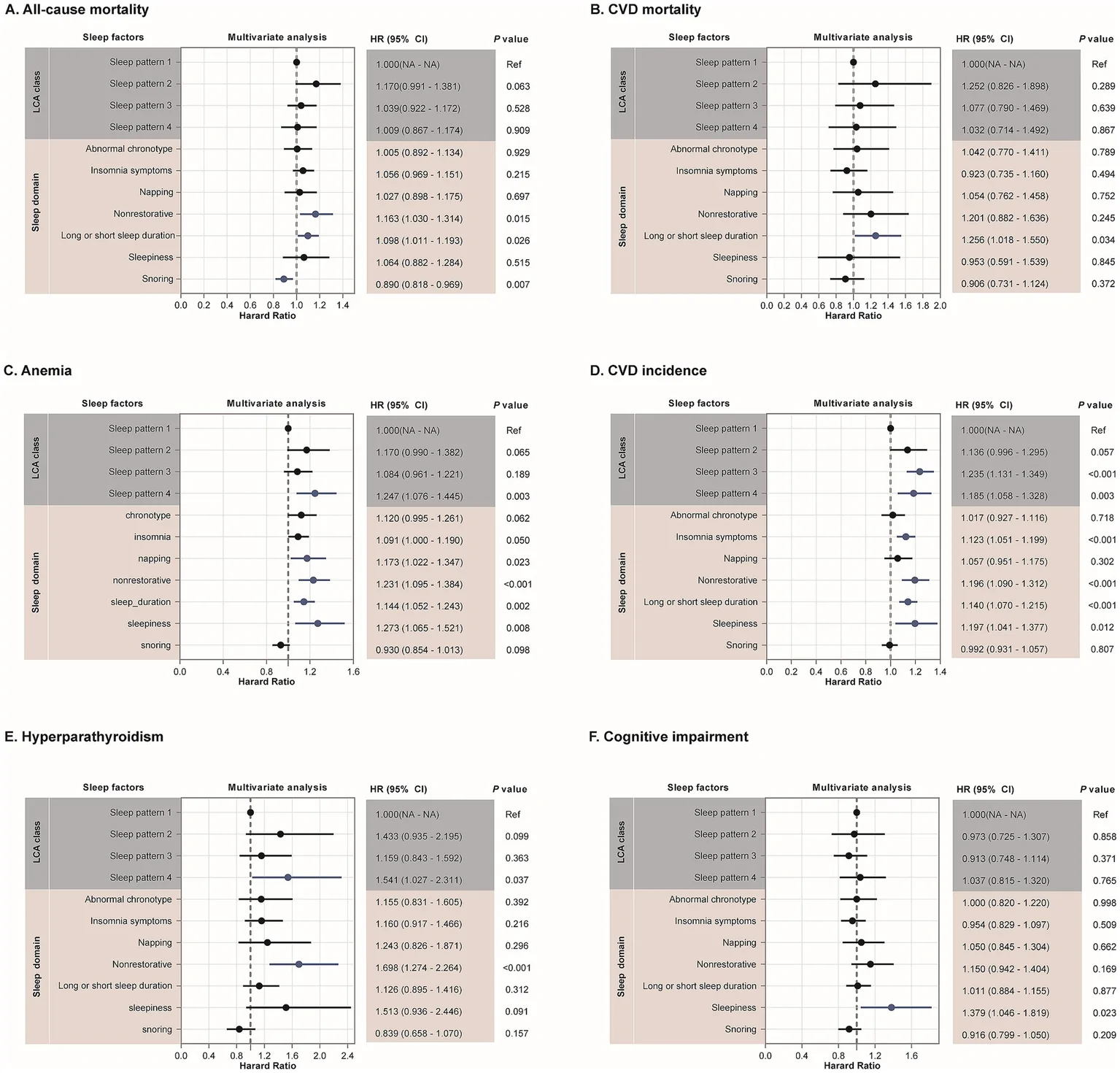
Forest plot of associations between sleep health factors and **(A)** all-cause mortality, **(B)** CVD mortality, **(C)** anemia, **(D)** CVD incidence, **(E)** hyperparathyroidism, and **(F)** cognitive impairment. CVD, cardiovascular disease.

**Figure 4:**
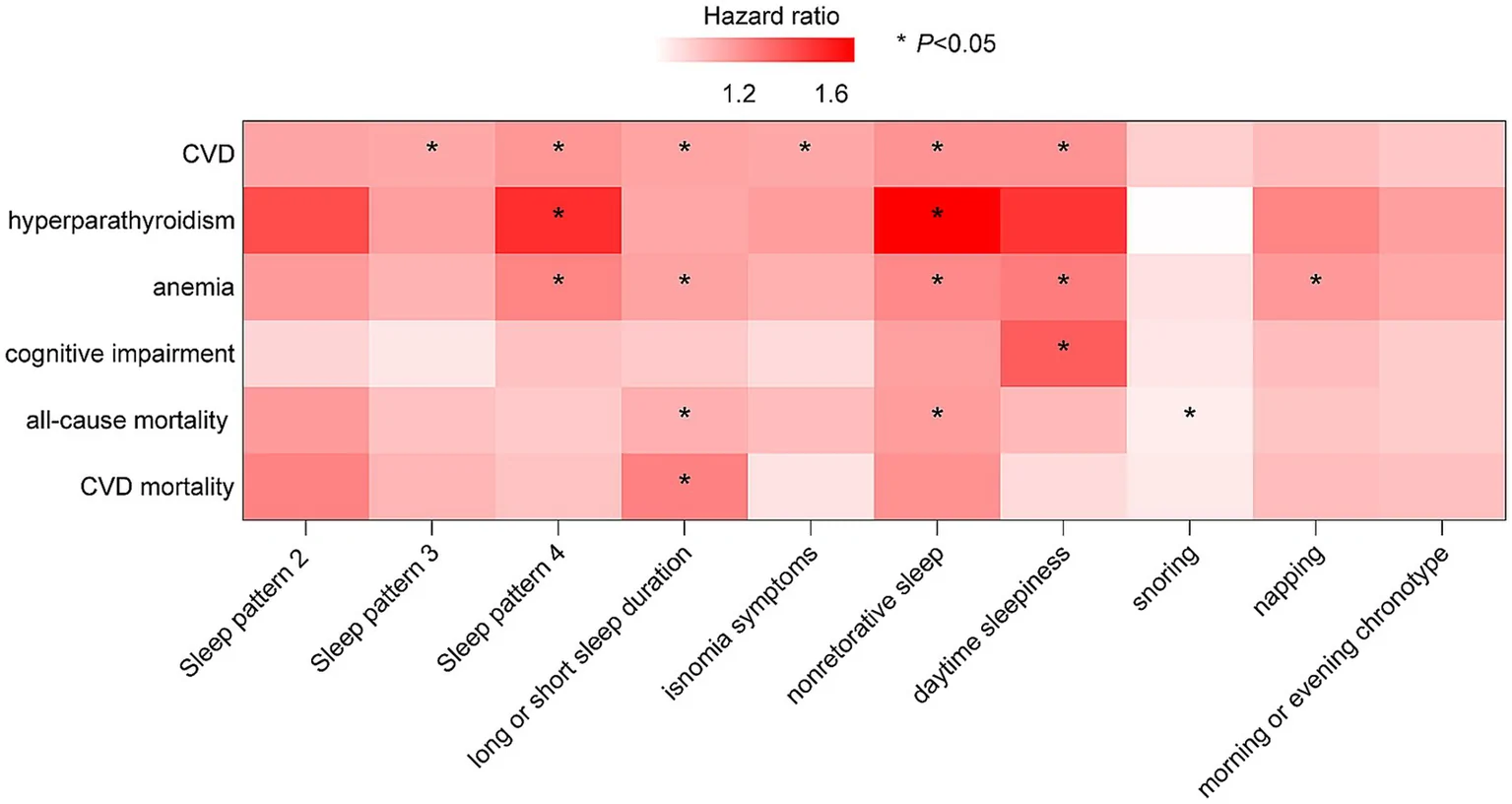
Heatmap of associations between different sleep health patterns, individual sleep factors, and health outcomes in the CKD population. The color red represents the intensity of association, and an asterisk represents statistical significance. CVD, cardiovascular diseases.

The average posterior probabilities were all >0.7 for the four sleep clusters (0.93, 0.92, 0.78, 0.74, respectively; Supplementary Table S6), indicating high reliability and distinguished discrimination of inter-cluster differences. As shown in Figures 3, 4, compared with the relatively healthy sleep cluster, sleep pattern 3 significantly aggravated risks for CVD incidence (HR, 1.235, 95%CI, 1.131–1.349, *p* < 0.001), severe sleep dysfunction (sleep pattern 4) associated with higher risks for CVD (HR, 1.185, 95%CI, 1.058–1.328, *p* = 0.003), hyperparathyroidism (HR, 1.541, 95%CI, 1.027–2.311, *p* = 0.037), and anemia (HR, 1.247, 95%CI, 1.076–1.445, *p* = 0.003). On the contrary, sleep pattern 2 demonstrated no associations with CKD-related complications and mortalities.

### 3.5 Each sleep factor in association with complications in the CKD population

To further understand how sleep factors contributed to the correlations between SHS, sleep clusters, and complications in CKD, we conducted a secondary analysis to evaluate the impact of individual sleep factors. As for mortality outcomes, non-restorative sleep and abnormal sleep duration intensified 10–20% risks for death, while abnormal sleep duration further aggravated risks of CVD mortality by approximately 25% (Figures 3A,B). Notably, snoring indicated a 10% decrease in risk for all-cause mortality. Risks of anemia and CVD were increased by insomnia symptoms, non-restorative sleep, abnormal sleep duration, and daytime sleepiness, with observed 10–25% risks increment (Figures 3C,D). Additionally, frequent naps also demonstrate a role in increasing the incidence of anemia (HR, 1.173, 95%CI, 1.022–1.374; Figure 3C). The association between sleep factors and hyperparathyroidism was stronger but inconsistent; only non-restorative sleep was linked to approximately 70% higher risks of hyperparathyroidism onset (Figure 3E). The risks for cognitive impairment were exclusively elevated by daytime sleepiness, with a 38% increment being observed (Figure 3F). Abnormal chronotypes (both morning and evening), when examined individually, were not associated with elevated risks of mortality and complications in the CKD population (Figure 4).

### 3.6 Subgroup analysis and sensitivity analysis

To understand whether sex dimorphism and preserved kidney function could potentially influence the pathway from sleep dysfunction to outcomes, we performed subgroup analysis based on sex (male and female) and CKD stages stratified by eGFR. Gender factors were significantly found to interact between sleep patterns and all-cause mortality, with females more vulnerable from sleep pattern 2 than males (*p* for interaction = 0.02; Supplementary Table S7). Sleep pattern 2 was correlated to higher risks of all-cause mortality in the lower eGFR group (*p* for interaction = 0.018, eGFR < 60; Supplementary Table S8). Although all sleep patterns failed to significantly associate with elevated risks of cognitive impairment, sleep pattern 4 was linked to a 62% higher risk of cognitive impairment in patients with 60 ≤ eGFR≤89 (*p* for interaction = 0.013; Supplementary Table S8). As for individual sleep factors, non-restorative sleep displayed significant sex dimorphism in all-cause mortality, CVD mortality, CVD incidence, and anemia (*p* for interaction = 0.007, 0.021, 0.041, 0.01, respectively; Supplementary Table S9), with higher risks in female patients than male. Moreover, female patients were also found to have higher risks of abnormal chronotype-associated all-cause mortality and long- or short-sleep duration-associated CVD incidence (both *p* for interaction = 0.007; Supplementary Table S9). The interactions of preserved kidney function were only observed in relationships between non-restorative sleep and all-cause mortality, with higher risks in poorer kidney function (*p* for interaction = 0.05, respectively; Supplementary Table S10).

Sensitivity analysis results excluded outcome events that happened in the first 3 years and further adjusted for depression and anxiety levels. The overall results of sensitivity analysis aligned with our previous findings, indicating robust relationships between multi-dimensional sleep dysfunction and poorer prognosis in the CKD population (Supplementary Tables S11,12).

## 4 Discussion

Sleep disorders exist in patients with or without CKD, and the possible characteristics and interventions for sleep disorders identified in the general population might not be applicable in CKD patients. Emerging sleep disorders are quite prevalent in CKD and associated with multiple poor prognoses, including CVD, worse kidney function, and lower life quality, warranting attention for patient-centered management (8, 9). In the present study, the multi-dimensional sleep health, categorized by simple SHS or sleep clusters, was correlated with incident CVD, anemia, and hyperparathyroidism after adjusting for a variety of covariates. LCA sleep clusters, especially the severely disturbed sleep (sleep pattern 4), demonstrated significant associations with higher risks for CVD, anemia, and hyperparathyroidism, indicating the complex effects of multi-dimensional sleep disturbances and sleep factors.

From 2010 to 2022, the American Heart Association updated its framework for the essential components impacting cardiovascular, renal, and metabolic health from the “Life’s Simple 7” to “Life’s Essential 8.” This transformation was primarily driven by the inclusion of sleep health, which was recognized as a crucial factor in this updated conclusion (24, 25). This change further underscores the consistent conclusions from experimental and population studies regarding the association between sleep health and adverse cardiovascular or renal outcomes. Much of the research focuses on sleep duration, overlooking the overlapping components, including duration, circadian rhythm, regularity, efficiency, satisfaction, and influences on daytime functions (26). These components may impact each other, and the degree of clustering varied based on age and sex, making it hard to define in research. As our SHS analysis demonstrated, a simple linear addition of unhealthy sleep factors was significantly associated with higher risks of CVD, anemia, and hyperparathyroidism in a dose–response pattern, which was consistent with findings from the general population (17, 27, 28). Additionally, age tendencies and sex differences were prominent in CKD patients. Consequently, exclusive exploration of single sleep health factors without consideration of other related variables might lead to variations in the direction and strength of associations across studies.

LCA analysis captured heterogeneities in sleep phenotypes in CKD patients. For instance, we identified sleep pattern 2 (serious chronotype disturbance, non-restorative sleep together with moderate insomnia) and sleep pattern 3 (prominent insomnia with severe abnormal sleep duration) as unique sleep paradigms in CKD. Sleep pattern 3 was the most prevalent sleep dysregulation in the CKD population, identifying a wide influence of insomnia symptoms. Additionally, sleep pattern 3 was found to be significantly associated with incident CVD, which aligned with previous investigations (29–31), as insomnia affects 30–67% of CKD individuals (8). Since CVD is a major cause of mortality among CKD patients, it is crucial for nephrologists and patients to focus on lifestyle management targeting insomnia-oriented sleep to mitigate the risk of adverse events. Sleep pattern 4 identifies a cluster of moderate-to-severe disturbances across various sleep factors, which is closely associated with anemia, hyperparathyroidism, and CVD in CKD patients. Previous research has primarily demonstrated sleep disturbances in CKD patients in different aspects, including insomnia symptoms, restless legs syndrome, and sleep apnea (8, 9, 32). However, sleep pattern 4, characterized by broad sleep impairments, has not been recognized in prior studies. These patients often exhibit a more perilous clinical profile, underscoring the synergistic effects of multiple sleep dysfunctions, especially for the occurrence of anemia and CVD. Notably, a 24% higher risk of CVD was observed in sleep cluster 3, while a 19% increased risk was observed in cluster 4, which may be attributable to the well-established vascular pathology of sleep apnea (33, 34). The associations with hyperparathyroidism demanded cautious interpretation. Due to the relatively small number of incident events observed during follow-up, the effects of certain sleep factors may have been obscured, warranting validation and extension of these findings in future long-term disease-specific prospective cohorts. Interestingly, sleep pattern 2 failed to aggregate risk profiles in the CKD population. These non-significant associations suggested that differences in chronotype may not confer substantial harm, but demanded further validation.

Furthermore, in terms of individual sleep factors, risks of CVD and anemia might be intensified by long or short sleep duration, non-restorative sleep and daytime sleepiness, while napping also played a role in increasing risks of anemia. Our results aligned with findings from diabetes or CVD populations (35–37). These adverse effects may stem from increased chronic stress levels, disturbed hormone hemostasis, and further impairment of kidney function due to physiological sleep deprivation (38–40). Similarly, daytime sleepiness was the only sleep factor that exhibited escalating risks for cognitive impairment, probably owing to its inherent damage to cognitive function from low-quality sleep (41). Additionally, psychological issues, including depression or anxiety, in CKD patients might exacerbate sleepiness, thus exerting harmful effects on cognitive function (42). Notably, sleep chronotype has not been effectively linked with CKD-related health outcomes, despite substantial research indicating that disruptions in sleep rhythm can increase the risk of adverse cardiovascular, neurological, and malignant events (43, 44). The simple classification of sleep chronotypes into “morning,” “evening,” and “intermediate” may lack accuracy, and the role of genetic predisposition to sleep rhythm disturbances remains unclear and warrants further investigation (45). Prevalence of snoring exhibited a negative association with all-cause mortality (46). Prior studies have shown conflicting results regarding the association between snoring and all-cause mortality, with a meta-analysis indicating no definitive correlation. Some residual confounders, including misclassification of snoring reports or the timepoint for treatment seeking, may obscure the exact association. Especially, as CKD patients are exposed to medical assistance more frequently than the general population, this decreased association of snoring with all-cause mortality might be linked with increased screening and awareness by healthcare providers in response to these symptoms. However, this conclusion warrants careful consideration, and further rigorous research is necessary to elucidate the underlying mechanisms and validate these findings.

Our subgroup analysis demonstrated that sex dimorphism may play a role in pathways from LCA clusters, individual sleep factors, to all-cause mortality, CVD mortality, CVD and anemia, with female patients more vulnerable than males. The protective roles of estrogen and estrogen analogs in female kidneys might account for the difference (47). In this study, most CKD patients have reached perimenopausal age, during which the physiological loss of estrogen and its analogs may exacerbate renal dysfunction, which could contribute to the observed gender differences in the associations between LCA clusters, individual sleep factors and CKD-related health outcomes. Residual kidney function demonstrated significant interactions with LCA clusters, non-restorative sleep and insomnia. Higher risks of adverse CKD outcomes were displayed in diminished kidney function subgroups. These findings warrant sustainable attention for clinical sleep healthcare in female patients with poorer kidney function.

Our study supports the feasibility of sleep-based behavioral intervention as a practical approach to prevent the incidence of adverse outcomes in the CKD population. Sleep alignment represents modifiable and measurable sleep phenotypes, including self-multi-dimensional sleep evaluation and essential circadian synchronization. At the individual level, patients could enhance daytime physical activity, maximize natural light exposure, and keep regular sleep–wake cycles. These interventions are cost-effective, non-invasive, and effective, making them feasible for broad propagation. Additionally, wide utilization of wearable devices provides continuous, objective monitoring of circadian patterns and sleep exposure metrics. Integration of this automatic sleep surveillance into a personal health tracking system may alarm early sleep disturbance and facilitate timely personalized feedback with practical lifestyle guidance, providing actionable targets for improving the prognosis of the CKD population.

To our knowledge, this study has the following innovations. First, the use of LCA and SHS identified characteristics of sleep dysfunction in CKD and considered sleep health as an integrative perspective encompassing multiple sleep-related factors. Beyond these, based on prospective follow-up data in the UK Biobank, this study reported the associations between multi-dimensional sleep health and CKD-related health outcomes in a systematic manner. However, this study has some inevitable limitations. First, self-reported sleep assessment is the first line in clinical evaluations of diagnosis and subsequent interventions. The heterogeneity of the self-reported data, as shown in this study, might lead to some misclassifications of sleep dysfunction and ignorance of some potential patients with sleep disorders. Second, these assessments do not encompass other objective parameters, including hypotonic burden (48) and slow-wave sleep (49), to evaluate the severity of these symptoms. Nevertheless, our multi-dimensional sleep health patterns based on these simple assessments are assessable in clinical management. Moreover, the sleep exposure was obtained from a single sleep assessment performed in a middle-aged CKD population. Although this single-time assessment showed moderate reliability and stability, it failed to fully capture the dynamic change of sleep over time, which should be sustained in future investigations (50). Finally, the sampling methods in the UK Biobank cohort overrepresent white participants with higher socioeconomic status, which may hinder the interpretation of these results to the general UK population and other races (51).

In general, poor sleep health assessed via a multi-dimensional framework was associated with higher risks of adverse outcomes in the CKD population. Considerable heterogeneity exists in sleep characteristics in the CKD population and their associations with adverse outcomes. Identifying the CKD-oriented sleep paradigm contributes to pinpointing subgroups at high risk and providing strategies for targeted interventions.

## Data Availability

Publicly available datasets were analyzed in this study. This data can be found at: The data used to support this study is available from UK Biobank website, subject to the registration and application procedure. Details could be found at: https://www.ukbiobank.ac.uk.
