# Supplementary material for: Multi-dimensional sleep patterns predict complications and mortality in chronic kidney disease patients: a prospective study from the UK Biobank
Source: Front Public Health. 2026 Aug 18;14:1889689. doi: 10.3389/fpubh.2026.1889689 (PMC13529563; doi:10.3389/fpubh.2026.1889689)
Supplement: Supplementary file 1 [file Table_1.docx]

Supplementary Methods

Definition of covariates

In our Cox multivariate analysis, included age, sex, race, disease history including hypertension and diabetes, body mass index (BMI), Townson’s deprivation index, lifestyle factors including smoking and alcohol consumption as well as history of taking RAASi drugs. Demographic information and Townson’s deprivation index were documented in baseline characteristics section via a questionnaire of since the first participance during 2006 to 2010. Detail information about smoking and drinking were obtained via a touchscreen questionnaire provided in examine center during first participance from 2006 to 2010. We considered all degrees of current smokers and drinkers as presence of history of smoking and drinking. BMI was calculated though body weight (kg)^^2^/height(m), with these parameters obtained in physical assessment centers. We considered patients with reports on either self-report disease history or International Coding Disease 10 (ICD-10) as diabetic or hypertensive patients. The ICD-10 code used for diabetes and hypertension were E10-14, I10-15 respectively. As for history of taking RAASi agents, we solely considered medication of renin-angiotensin-aldosterone system inhibitors due to its prevalent use in CKD population, which was obtained through an interactive touchscreen questionnaire.

Furthermore, in our sensitivity analysis, we additionally included presence of anxiety and depression into adjustment. Similarly, a patient was defined as depressed or anxious based on either self-reports or ICD-10. The ICD-10 used for classification of depression and anxiety was F41 or F32 respectively.

Supplementary Figures:

Figure S1. Distribution trend of scores of each sleep factor and SHS in different age groups. SHS, sleep health score.

Supplementary Tables

Table S1. Definitions used for identifications of chronic kidney disease patients.

| The definition criteria | Definitions^a^ |
| --- | --- |
| CKD-EPI 2021 | eGFR < 60 ml/min/1.73 m^2^ |
| Urine examination | UACR >30 mg/g |
| ICD-10 | N03, N11, N18 |
| Office of Population Censuses and Surveys Classification of Interventions and Procedures-version 4 | L74.1–74.6, L74.8–74.9, M01.2–01.9, M02.3, M08.4, M17.2, M17.4, M17.8–17.9, X40.1–40.9, X41.1–41.2, X41.8–41.9, X42.1, X42.8–42.9 |

a. Chronic kidney disease was identified as reaching either one of the above criteria. CKD-EPI, the Chronic Kidney Disease Epidemiology Collaboration equation; eGFR, estimated glomerular filtration rate; UACR, urinary albumin creatinine ratio; ICD-10, international code disease.

| Table S2. Assessment of self-reported sleep health factors^1^ | | | |
| --- | --- | --- | --- |
| Sleep health dimensions | Data field ID | Assessment questions | Responses and categorization^2^ |
| Sleep duration^3^ | 1160 | About how many hours sleep do you get in every 24 hours? (please include naps) | <7 hours (short) versus 7-8 hours (medium) versus >8 hours (long) |
| Insomnia symptoms | 1200 | Do you have trouble falling asleep at night or do you wake up in the middle of the night? | "Usually" versus "Sometimes", "Never/rarely" |
| Non-restorative sleep | 1170 | On an average day, how easy do you find getting up in the morning? | "Not at all easy", "Not very easy" versus "Fairly easy", "Very easy" |
| Daytime sleepiness | 1220 | How likely are you to doze off or fall asleep during the daytime when you don't mean to? (e.g., when working, reading or driving) | "Often" versus "Sometimes", "Never/rarely" |
| Snoring | 1210 | Does your partner or a close relative or friend complain about your snoring? | "Yes" versus "No" |
| Napping | 1190 | Do you have a nap during the day? | "Usually" versus "Sometimes", "Never/rarely" |
| Chronotype | 1180 | Do you consider yourself to be? | "Definitely a 'morning' person" (morning chronotype) versus "More a 'morning' than 'evening' person", "More an 'evening' than 'morning' person" (intermediate chronotype) versus "Definitely an ‘evening' person (evening chronotype) |
| ^1^Link to UK Biobank Showcase: <https://biobank.ndph.ox.ac.uk/showcase/label.cgi?id=100057> | | | |
| ^2^Participants who selected responses such as “prefer not to answer”, “do not know” or who skipped the question were classified as having missing data for that item and were excluded from the analysis | | | |
| ^3^Participants who reported sleep duration <3 hours or >14 hours were excluded from the analysis | | | |

Table S3. ICD-10 clinical diagnosis used for identification of outcome events

| Outcome | ICD-10 code |
| --- | --- |
| Anemia | D50-D64 |
| CVD | I25, I20, I21, I22, I24, I50, I48, I70, I74, I77, I60, I61, I63, I64 |
| Cognitive impairment | F06.7, G31.8, R41, F00, F01, F02, F03, G30 |
| Hyperparathyroidism | E21, N25.0, M85.8 |

Table S4. The association of different SHS categories and all-cause mortality in CKD population

| Outcomes | SHS category | Events | HR (95%CI)^a^ | *P* value |
| --- | --- | --- | --- | --- |
| All-cause mortality | Healthiest | 1299 | 1.000 | Ref. |
|  | Mild | 663 | 1.111 (1.011 - 1.221) | 0.028 |
|  | Moderate | 406 | 1.041 (0.930 - 1.167) | 0.483 |
|  | Severe | 48 | 1.241 (0.927 - 1.660) | 0.147 |
| CVD mortality | Healthiest | 182 | 1.000 | Ref. |
|  | Mild | 110 | 1.258 (0.991 - 1.596) | 0.059 |
|  | Moderate | 63 | 1.056 (0.789 - 1.415) | 0.713 |
|  | Severe | 7 | 1.067 (0.497 - 2.290) | 0.868 |
| Anemia | Healthiest | 1232 | 1.000 | Ref. |
|  | Mild | 630 | 1.124 (1.020 - 1.238) | 0.018 |
|  | Moderate | 431 | 1.191 (1.064 - 1.332) | 0.002 |
|  | Severe | 50 | 1.418 (1.065 - 1.887) | 0.017 |
| Cognitive impairment | Healthiest | 551 | 1.000 | Ref. |
|  | Mild | 214 | 0.864 (0.737 - 1.012) | 0.071 |
|  | Moderate | 161 | 1.065 (0.890 - 1.273) | 0.493 |
|  | Severe | 14 | 0.998 (0.585 - 1.704) | 0.995 |
| CVD | Healthiest | 2151 | 1.000 | Ref. |
|  | Mild | 1092 | 1.114 (1.035 - 1.198) | 0.004 |
|  | Moderate | 775 | 1.260 (1.159 - 1.370) | < 0.001 |
|  | Severe | 69 | 1.128 (0.886 - 1.437) | 0.328 |
| Hyperparathyroidism | Healthiest | 171 | 1.000 | Ref. |
|  | Mild | 70 | 0.983 (0.742 - 1.301) | 0.903 |
|  | Moderate | 69 | 1.600 (1.202 - 2.130) | 0.001 |
|  | Severe | 4 | 1.078 (0.398 - 2.922) | 0.883 |

a. The model used to investigate the association of SHS and complications in CKD was fully adjusted for age, sex, races, diabetes, hypertension, BMI, Townson’s deprivation index, smoking, drinking and history of taking RAASi agents.

Abbreviations: CKD, chronic kidney disease; CVD, cardiovascular diseases; SHS, sleep health score; HR, hazard ratio.

Table S5: Parameters for model selection of LCA analysis

| Numbers of clusters | BIC | AIC | Gsq | logLik |
| --- | --- | --- | --- | --- |
| 2 | 95351.07 | 95236.05 | 1602.21 | -47603.02 |
| 3 | 94501.56 | 94325.19 | 675.36 | -47139.59 |
| 4 | 94154.69 | 93916.98 | 251.15 | -46927.49 |
| 5 | 94156.22 | 93957.16 | 175.33 | -46889.58 |
| 6 | 94183.74 | 93823.33 | 125.49 | -46864.66 |

Table S6: Average posterior probabilities of 4 clusters identified by LCA analysis

| Cluster | Sample sizes | Posterior probabilities | Standard error of posterior probabilities |
| --- | --- | --- | --- |
| Sleep pattern 1 | 11896 | 92.6% | 0.11 |
| Sleep pattern 2 | 952 | 91.7% | 0.04 |
| Sleep pattern 3 | 2016 | 78.9% | 0.08 |
| Sleep pattern 4 | 945 | 73.8% | 0.16 |

Table S7. Subgroup analysis to explore interactive factors of gender on pathways from LCA sleep clusters and outcomes

| Outcomes | LCA sleep clusters ^a^ | | | | *P* for interaction |
| --- | --- | --- | --- | --- | --- |
|  | Sleep pattern 1 | Sleep pattern 2 | Sleep pattern 3 | Sleep pattern 4 |  |
|  |  |  |  |  |  |
| **All-cause mortality** |  |  |  |  |  |
| female | 1.00 (ref) | 1.388 (1.105 - 1.742) | 0.901 (0.751 - 1.081) | 0.990 (0.750 - 1.305) | 0.020 |
| male | 1.00 (ref) | 0.977 (0.766 - 1.245) | 1.171 (0.999 - 1.373) | 1.021 (0.852 - 1.224) |  |
|  |  |  |  |  |  |
| **CVD mortality** |  |  |  |  |  |
| female | 1.00 (ref) | 1.925 (1.060 - 3.495) | 1.103 (0.669 - 1.817) | 0.568 (0.206 - 1.563) | 0.179 |
| male | 1.00 (ref) | 0.907 (0.503 - 1.637) | 1.047 (0.702 - 1.562) | 1.178 (0.790 - 1.758) |  |
|  |  |  |  |  |  |
| **CVD incidence** |  |  |  |  |  |
| female | 1.00 (ref) | 1.169 (0.965 - 1.416) | 1.339 (1.183 - 1.515) | 1.358 (1.119 - 1.647) | 0.096 |
| male | 1.00 (ref) | 1.116 (0.932 - 1.337) | 1.145 (1.008 - 1.301) | 1.123 (0.975 - 1.293) |  |
|  |  |  |  |  |  |
| **Hyperparathyroidism** |  |  |  |  |  |
| female | 1.00 (ref) | 1.269 (0.742 - 2.169) | 1.255 (0.878 - 1.795) | 1.533 (0.898 - 2.617) | 0.518 |
| male | 1.00 (ref) | 1.923 (0.947 - 3.905) | 0.919 (0.456 - 1.854) | 1.511 (0.805 - 2.839) |  |
|  |  |  |  |  |  |
| **Cognitive impairment** |  |  |  |  |  |
| female | 1.00 (ref) | 1.223 (0.839 - 1.784) | 0.824 (0.622 - 1.092) | 0.862 (0.558 - 1.331) | 0.221 |
| male | 1.00 (ref) | 0.742 (0.461 - 1.196) | 1.023 (0.772 - 1.356) | 1.157 (0.865 - 1.549) |  |
|  |  |  |  |  |  |
| **Anemia** |  |  |  |  |  |
| female | 1.00 (ref) | 1.205 (0.970 - 1.499) | 1.019 (0.868 - 1.196) | 1.336 (1.061 - 1.681) | 0.393 |
| male | 1.00 (ref) | 1.095 (0.843 - 1.421) | 1.170 (0.977 - 1.401) | 1.208 (0.997 - 1.464) |  |

a. The model used in subgroup analysis was fully adjusted for age, sex, races, diabetes, hypertension, BMI, Townson’s deprivation index, smoking, drinking and history of taking RAASi agents.

Abbreviations: CVD, cardiovascular disease; LCA, latent class analysis

Table S8. Subgroup analysis to explore interactive factors of residual kidney function on pathways from LCA sleep clusters and outcomes

| Outcomes^a^ | LCA sleep cluster ^b^ | | | | *P* for interaction |
| --- | --- | --- | --- | --- | --- |
|  | Sleep pattern 1 | Sleep pattern 2 | Sleep pattern 3 | Sleep pattern 4 |  |
|  |  |  |  |  |  |
| **All-cause mortality** |  |  |  |  |  |
| <45 | 1.00 (ref) | 2.324 (1.404 - 3.844) | 1.097 (0.696 - 1.727) | 1.099 (0.624 - 1.936) | 0.018 |
| 45-59 | 1.00 (ref) | 1.513 (1.061 - 2.157) | 0.964 (0.733 - 1.267) | 0.896 (0.627 - 1.280) |  |
| 60-89 | 1.00 (ref) | 1.154 (0.843 - 1.580) | 0.896 (0.711 - 1.130) | 1.034 (0.800 - 1.337) |  |
| >90 | 1.00 (ref) | 0.852 (0.621 - 1.170) | 1.208 (0.990 - 1.473) | 1.028 (0.783 - 1.349) |  |
|  |  |  |  |  |  |
| **CVD mortality** |  |  |  |  |  |
| <45 | 1.00 (ref) | 1.383 (0.304 - 6.294) | 1.700 (0.659 - 4.383) | 0.760 (0.201 - 2.868) | 0.977 |
| 45-59 | 1.00 (ref) | 1.706 (0.770 - 3.780) | 0.816 (0.402 - 1.656) | 0.941 (0.424 - 2.089) |  |
| 60-89 | 1.00 (ref) | 1.218 (0.557 - 2.663) | 1.130 (0.657 - 1.944) | 1.074 (0.579 - 1.990) |  |
| >90 | 1.00 (ref) | 1.212 (0.579 - 2.539) | 0.940 (0.520 - 1.699) | 0.780 (0.355 - 1.714) |  |
|  |  |  |  |  |  |
| **CVD incidence** |  |  |  |  |  |
| <45 | 1.00 (ref) | 1.112 (0.672 - 1.841) | 1.278 (0.877 - 1.861) | 1.595 (1.038 - 2.450) | 0.339 |
| 45-59 | 1.00 (ref) | 1.073 (0.787 - 1.462) | 1.237 (1.019 - 1.501) | 1.103 (0.851 - 1.428) |  |
| 60-89 | 1.00 (ref) | 1.069 (0.826 - 1.383) | 1.309 (1.115 - 1.535) | 1.315 (1.086 - 1.592) |  |
| >90 | 1.00 (ref) | 1.120 (0.905 - 1.387) | 1.151 (0.984 - 1.345) | 1.000 (0.808 - 1.238) |  |
|  |  |  |  |  |  |
| **Hyperparathyroidism** |  |  |  |  |  |
| <45 | 1.00 (ref) | 1.548 (0.436 - 5.494) | 1.157 (0.395 - 3.394) | 2.277 (0.882 - 5.880) | 0.079 |
| 45-59 | 1.00 (ref) | 1.440 (0.565 - 3.673) | 1.519 (0.814 - 2.833) | 0.747 (0.229 - 2.432) |  |
| 60-89 | 1.00 (ref) | 1.051 (0.422 - 2.619) | 0.336 (0.135 - 0.835) | 1.378 (0.656 - 2.892) |  |
| >90 | 1.00 (ref) | 1.851 (0.869 - 3.943) | 1.929 (1.139 - 3.268) | 1.436 (0.612 - 3.370) |  |
|  |  |  |  |  |  |
| **Cognitive impairment** |  |  |  |  |  |
| <45 | 1.00 (ref) | 0.817 (0.244 - 2.739) | 0.682 (0.259 - 1.800) | 0.799 (0.238 - 2.685) | 0.013 |
| 45-59 | 1.00 (ref) | 1.141 (0.617 - 2.110) | 1.149 (0.787 - 1.678) | 0.433 (0.202 - 0.925) |  |
| 60-89 | 1.00 (ref) | 0.733 (0.387 - 1.388) | 0.584 (0.372 - 0.917) | 1.615 (1.132 - 2.305) |  |
| >90 | 1.00 (ref) | 0.850 (0.509 - 1.419) | 0.887 (0.625 - 1.258) | 1.024 (0.662 - 1.585) |  |
|  |  |  |  |  |  |
| **Anemia** |  |  |  |  |  |
| <45 | 1.00 (ref) | 1.082 (0.621 - 1.885) | 1.064 (0.690 - 1.638) | 1.533 (0.973 - 2.415) | 0.411 |
| 45-59 | 1.00 (ref) | 1.582 (1.123 - 2.229) | 1.127 (0.870 - 1.460) | 1.100 (0.784 - 1.544) |  |
| 60-89 | 1.00 (ref) | 1.143 (0.818 - 1.595) | 0.972 (0.771 - 1.227) | 1.348 (1.041 - 1.745) |  |
| >90 | 1.00 (ref) | 0.887 (0.649 - 1.210) | 1.180 (0.958 - 1.454) | 1.218 (0.926 - 1.601) |  |

a. Residual kidney function was assessed through eGFR calculated via CKD-EPI 2021

b. The model used in subgroup analysis was fully adjusted for age, sex, races, diabetes, hypertension, BMI, Townson’s deprivation index, smoking, drinking and history of taking RAASi agents.

Abbreviations: CVD, cardiovascular disease; LCA, latent class analysis

Table S9. Subgroup analysis to explore interactive factors of gender on pathways from individual sleep factors and outcomes

| Outcomes | Sleep factors ^a^ | | | | | | |
| --- | --- | --- | --- | --- | --- | --- | --- |
|  | Long or short sleep duration | Insomnia | Nonrestorative | Daytime sleepiness | Snoring | Napping | Early or evening chronotype |
| **All-cause mortality** |  |  |  |  |  |  |  |
| female | 1.011 (0.887 - 1.152) | 0.967 (0.847 - 1.104) | 1.374 (1.161 - 1.626) | 1.060 (0.768 - 1.463) | 0.985 (0.859 - 1.129) | 1.074 (0.825 - 1.398) | 1.216 (1.016 - 1.454) |
| male | 1.163 (1.045 - 1.294) | 1.126 (1.005 - 1.261) | 0.983 (0.823 - 1.173) | 1.066 (0.846 - 1.343) | 0.839 (0.754 - 0.933) | 1.014 (0.867 - 1.186) | 0.874 (0.744 - 1.028) |
| P for interaction | 0.114 | 0.123 | 0.007 | 0.968 | 0.058 | 0.613 | 0.007 |
|  |  |  |  |  |  |  |  |
| **CVD mortality** |  |  |  |  |  |  |  |
| female | 1.477 (1.017 - 2.145) | 0.850 (0.573 - 1.262) | 1.840 (1.170 - 2.894) | 0.820 (0.300 - 2.242) | 0.922 (0.613 - 1.387) | 1.237 (0.619 - 2.469) | 1.563 (0.966 - 2.527) |
| male | 1.166 (0.903 - 1.505) | 0.951 (0.719 - 1.259) | 0.875 (0.566 - 1.352) | 1.012 (0.587 - 1.746) | 0.889 (0.689 - 1.146) | 1.037 (0.718 - 1.498) | 0.834 (0.563 - 1.235) |
| P for interaction | 0.215 | 0.969 | 0.021 | 0.835 | 0.945 | 0.393 | 0.043 |
|  |  |  |  |  |  |  |  |
| **CVD incidence** |  |  |  |  |  |  |  |
| female | 1.250 (1.135 - 1.377) | 1.172 (1.063 - 1.293) | 1.350 (1.187 - 1.536) | 1.439 (1.158 - 1.788) | 0.992 (0.896 - 1.099) | 1.068 (0.871 - 1.309) | 0.978 (0.845 - 1.133) |
| male | 1.061 (0.976 - 1.154) | 1.083 (0.990 - 1.184) | 1.072 (0.937 - 1.225) | 1.081 (0.900 - 1.298) | 0.989 (0.911 - 1.073) | 1.073 (0.948 - 1.215) | 1.042 (0.924 - 1.175) |
| P for interaction | 0.007 | 0.164 | 0.041 | 0.030 | 0.805 | 0.653 | 0.416 |
|  |  |  |  |  |  |  |  |
| **Hyperparathyroidism** |  |  |  |  |  |  |  |
| female | 1.124 (0.854 - 1.480) | 1.130 (0.855 - 1.493) | 1.761 (1.260 - 2.459) | 1.511 (0.820 - 2.784) | 0.806 (0.594 - 1.094) | 1.736 (1.053 - 2.861) | 1.223 (0.824 - 1.817) |
| male | 1.119 (0.736 - 1.701) | 1.258 (0.817 - 1.937) | 1.589 (0.903 - 2.795) | 1.489 (0.681 - 3.254) | 0.926 (0.611 - 1.402) | 0.764 (0.381 - 1.532) | 1.040 (0.574 - 1.884) |
| P for interaction | 0.774 | 0.406 | 0.916 | 0.833 | 0.342 | 0.104 | 0.923 |
|  |  |  |  |  |  |  |  |
| **Cognitive impairment** |  |  |  |  |  |  |  |
| female | 0.975 (0.800 - 1.187) | 0.962 (0.788 - 1.175) | 1.317 (1.010 - 1.718) | 1.421 (0.923 - 2.187) | 0.921 (0.746 - 1.137) | 0.893 (0.585 - 1.364) | 1.297 (0.988 - 1.703) |
| male | 1.032 (0.860 - 1.239) | 0.952 (0.781 - 1.159) | 1.005 (0.741 - 1.363) | 1.361 (0.948 - 1.953) | 0.910 (0.760 - 1.089) | 1.138 (0.882 - 1.468) | 0.786 (0.587 - 1.051) |
| P for interaction | 0.753 | 0.879 | 0.253 | 0.817 | 0.852 | 0.539 | 0.018 |
|  |  |  |  |  |  |  |  |
| **Anemia** |  |  |  |  |  |  |  |
| female | 1.085 (0.964 - 1.221) | 1.138 (1.010 - 1.281) | 1.356 (1.167 - 1.575) | 1.348 (1.037 - 1.752) | 0.890 (0.786 - 1.009) | 1.300 (1.034 - 1.634) | 1.162 (0.985 - 1.370) |
| male | 1.209 (1.073 - 1.362) | 1.034 (0.910 - 1.175) | 1.057 (0.875 - 1.277) | 1.216 (0.954 - 1.550) | 0.965 (0.857 - 1.085) | 1.114 (0.937 - 1.323) | 1.071 (0.902 - 1.271) |
| P for interaction | 0.331 | 0.181 | 0.010 | 0.423 | 0.857 | 0.233 | 0.284 |

a. The model used in subgroup analysis was fully adjusted for age, sex, races, diabetes, hypertension, BMI, Townson’s deprivation index, smoking, drinking and history of taking RAASi agents.

Table S10. Subgroup analysis to explore interactive factors of residual kidney function on pathways from individual sleep factors to outcomes

| Outcomes | Sleep factors | | | | | | |  |
| --- | --- | --- | --- | --- | --- | --- | --- | --- |
|  | Sleep duration score | Insomnia score | Nonrestorative score | Sleepiness score | Snoring score | Napping score | Chronotype score |  |
| All-cause mortality |  |  |  |  |  |  |  |  |
| <45 | 1.339 (0.986 - 1.818) | 1.177 (0.860 - 1.611) | 1.484 (0.987 - 2.231) | 0.665 (0.288 - 1.536) | 1.220 (0.880 - 1.693) | 1.113 (0.715 - 1.732) | 1.582 (1.056 - 2.371) |  |
| 45-59 | 1.083 (0.901 - 1.303) | 1.005 (0.828 - 1.219) | 1.341 (1.021 - 1.761) | 0.906 (0.563 - 1.460) | 0.888 (0.736 - 1.071) | 1.033 (0.763 - 1.399) | 1.069 (0.819 - 1.396) |  |
| 60-89 | 1.002 (0.861 - 1.166) | 0.956 (0.814 - 1.122) | 1.172 (0.930 - 1.477) | 1.110 (0.799 - 1.543) | 0.810 (0.696 - 0.944) | 1.017 (0.803 - 1.288) | 1.091 (0.878 - 1.356) |  |
| >90 | 1.119 (0.969 - 1.293) | 1.153 (0.993 - 1.339) | 1.045 (0.845 - 1.293) | 1.192 (0.881 - 1.613) | 0.877 (0.756 - 1.016) | 0.882 (0.680 - 1.144) | 0.794 (0.638 - 0.989) |  |
| P for interaction | 0.306 | 0.392 | 0.050 | 0.868 | 0.315 | 0.666 | 0.010 |  |
|  |  |  |  |  |  |  |  |  |
| CVD mortality |  |  |  |  |  |  |  |  |
| <45 | 1.103 (0.547 - 2.225) | 0.833 (0.391 - 1.774) | 1.558 (0.570 - 4.261) | 0.492 (0.056 - 4.301) | 0.749 (0.351 - 1.597) | 1.169 (0.455 - 3.003) | 1.000 (0.348 - 2.869) |  |
| 45-59 | 1.213 (0.780 - 1.887) | 0.889 (0.552 - 1.434) | 1.698 (0.943 - 3.058) | 0.943 (0.338 - 2.627) | 0.813 (0.515 - 1.285) | 1.232 (0.626 - 2.423) | 1.003 (0.514 - 1.957) |  |
| 60-89 | 1.105 (0.757 - 1.613) | 1.017 (0.682 - 1.515) | 1.031 (0.564 - 1.886) | 1.364 (0.657 - 2.829) | 0.954 (0.654 - 1.391) | 0.869 (0.473 - 1.596) | 1.109 (0.650 - 1.893) |  |
| >90 | 1.205 (0.815 - 1.780) | 0.732 (0.468 - 1.144) | 1.135 (0.647 - 1.992) | 0.467 (0.146 - 1.490) | 0.996 (0.668 - 1.484) | 0.862 (0.443 - 1.678) | 0.940 (0.539 - 1.639) |  |
| P for interaction | 0.951 | 0.480 | 0.476 | 0.331 | 0.882 | 0.861 | 0.970 |  |
|  |  |  |  |  |  |  |  |  |
| CVD incidence |  |  |  |  |  |  |  |  |
| <45 | 1.297 (1.000 - 1.681) | 1.109 (0.847 - 1.453) | 1.176 (0.831 - 1.663) | 1.893 (1.072 - 3.345) | 0.767 (0.581 - 1.012) | 1.332 (0.934 - 1.899) | 0.905 (0.603 - 1.357) |  |
| 45-59 | 1.045 (0.908 - 1.204) | 1.019 (0.879 - 1.180) | 1.308 (1.064 - 1.608) | 1.133 (0.815 - 1.575) | 0.997 (0.867 - 1.147) | 1.130 (0.899 - 1.419) | 0.998 (0.808 - 1.234) |  |
| 60-89 | 1.166 (1.039 - 1.307) | 1.265 (1.124 - 1.424) | 1.167 (0.978 - 1.392) | 1.160 (0.899 - 1.497) | 1.012 (0.902 - 1.136) | 1.077 (0.894 - 1.297) | 0.885 (0.741 - 1.058) |  |
| >90 | 1.102 (0.987 - 1.230) | 1.063 (0.947 - 1.194) | 1.126 (0.963 - 1.317) | 1.181 (0.933 - 1.494) | 1.044 (0.935 - 1.167) | 0.878 (0.716 - 1.075) | 1.107 (0.951 - 1.290) |  |
| P for interaction | 0.327 | 0.097 | 0.513 | 0.137 | 0.269 | 0.150 | 0.213 |  |
|  |  |  |  |  |  |  |  |  |
| Hyperparathyroidism |  |  |  |  |  |  |  |  |
| <45 | 1.300 (0.655 - 2.581) | 1.788 (0.904 - 3.538) | 1.316 (0.552 - 3.134) | 2.007 (0.579 - 6.960) | 0.810 (0.385 - 1.701) | 1.990 (0.849 - 4.666) | 1.554 (0.626 - 3.861) |  |
| 45-59 | 1.394 (0.855 - 2.271) | 1.519 (0.926 - 2.489) | 1.325 (0.668 - 2.632) | 0.765 (0.184 - 3.170) | 0.906 (0.540 - 1.521) | 1.036 (0.411 - 2.612) | 1.053 (0.499 - 2.220) |  |
| 60-89 | 0.876 (0.561 - 1.367) | 0.511 (0.303 - 0.861) | 1.670 (0.965 - 2.889) | 1.447 (0.581 - 3.602) | 0.788 (0.495 - 1.255) | 1.130 (0.485 - 2.631) | 1.201 (0.649 - 2.220) |  |
| >90 | 1.146 (0.741 - 1.770) | 1.383 (0.894 - 2.141) | 1.739 (1.021 - 2.961) | 1.171 (0.426 - 3.219) | 0.798 (0.499 - 1.275) | 0.960 (0.382 - 2.408) | 0.973 (0.498 - 1.901) |  |
| P for interaction | 0.589 | 0.007 | 0.954 | 0.773 | 0.575 | 0.356 | 0.870 |  |
|  |  |  |  |  |  |  |  |  |
| Cognitive impairment |  |  |  |  |  |  |  |  |
| <45 | 0.900 (0.489 - 1.656) | 1.147 (0.617 - 2.133) | 0.801 (0.311 - 2.064) | 0.500 (0.066 - 3.803) | 0.819 (0.418 - 1.606) | 1.159 (0.475 - 2.826) | 1.028 (0.429 - 2.465) |  |
| 45-59 | 0.901 (0.678 - 1.198) | 1.096 (0.823 - 1.459) | 1.311 (0.859 - 2.003) | 0.475 (0.176 - 1.283) | 0.866 (0.649 - 1.156) | 0.710 (0.410 - 1.227) | 1.267 (0.853 - 1.882) |  |
| 60-89 | 0.879 (0.682 - 1.132) | 0.850 (0.650 - 1.111) | 0.970 (0.646 - 1.458) | 2.078 (1.339 - 3.223) | 1.079 (0.841 - 1.384) | 1.625 (1.162 - 2.273) | 0.788 (0.524 - 1.185) |  |
| >90 | 1.087 (0.861 - 1.372) | 0.907 (0.707 - 1.163) | 1.210 (0.869 - 1.684) | 1.840 (1.212 - 2.794) | 0.838 (0.658 - 1.068) | 0.840 (0.544 - 1.296) | 0.848 (0.595 - 1.208) |  |
| P for interaction | 0.665 | 0.575 | 0.625 | 0.010 | 0.425 | 0.013 | 0.483 |  |
|  |  |  |  |  |  |  |  |  |
| Anemia |  |  |  |  |  |  |  |  |
| <45 | 1.208 (0.909 - 1.605) | 1.113 (0.832 - 1.489) | 1.139 (0.777 - 1.671) | 1.276 (0.692 - 2.353) | 0.961 (0.714 - 1.293) | 1.162 (0.776 - 1.740) | 0.954 (0.622 - 1.462) |  |
| 45-59 | 1.056 (0.881 - 1.267) | 1.105 (0.916 - 1.332) | 1.430 (1.110 - 1.841) | 1.049 (0.675 - 1.631) | 0.883 (0.733 - 1.064) | 1.225 (0.910 - 1.650) | 1.379 (1.075 - 1.768) |  |
| 60-89 | 1.243 (1.063 - 1.453) | 0.975 (0.826 - 1.152) | 1.172 (0.930 - 1.477) | 1.409 (1.024 - 1.939) | 1.034 (0.882 - 1.211) | 1.222 (0.953 - 1.565) | 1.267 (1.021 - 1.572) |  |
| >90 | 1.151 (0.990 - 1.337) | 1.150 (0.984 - 1.344) | 1.056 (0.857 - 1.300) | 1.334 (0.985 - 1.808) | 0.877 (0.752 - 1.023) | 1.126 (0.863 - 1.468) | 0.930 (0.747 - 1.158) |  |
| P for interaction | 0.486 | 0.503 | 0.410 | 0.702 | 0.440 | 0.803 | 0.087 |  |

a. Residual kidney function was assessed through eGFR calculated via CKD-EPI 2021

b. The model used in subgroup analysis was fully adjusted for age, sex, races, diabetes, hypertension, BMI, Townson’s deprivation index, smoking, drinking and history of taking RAASi agents.

Table S11. Sensitivity analysis after exclusion of outcomes happens within the first three years of follow-up

| Outcome | Exposure | Events | HR (95%CI) | P value |
| --- | --- | --- | --- | --- |
| All -cause mortality | SHS (continuous) | NA | 1.002 (0.964 - 1.043) | 0.908 |
|  | SHS-minima | 1020 | 1.000 (NA - NA) | Ref. |
|  | SHS-mild | 512 | 1.120 (1.006 - 1.246) | 0.0384 |
|  | SHS-moderate | 285 | 0.968 (0.847 - 1.106) | 0.6323 |
|  | SHS-severe | 36 | 1.199 (0.856 - 1.680) | 0.2905 |
|  | Sleep pattern 1 | 1361 | 1.000 (NA - NA) | Ref. |
|  | Sleep pattern 2 | 116 | 1.143 (0.944 - 1.385) | 0.171 |
|  | Sleep pattern 3 | 240 | 1.025 (0.893 - 1.177) | 0.725 |
|  | Sleep pattern 4 | 136 | 0.970 (0.811 - 1.159) | 0.734 |
|  | Abnormal chronotype | 241 | 1.014 (0.884 - 1.163) | 0.846 |
|  | Long or short sleep duration | 702 | 1.061 (0.965 - 1.167) | 0.219 |
|  | Insomnia | 573 | 1.012 (0.916 - 1.118) | 0.817 |
|  | Napping | 176 | 0.989 (0.845 - 1.159) | 0.895 |
|  | Snoring | 728 | 0.872 (0.792 - 0.960) | 0.005 |
|  | Nonrestorative | 223 | 1.104 (0.958 - 1.273) | 0.173 |
|  | Day time sleepiness | 84 | 1.035 (0.830 - 1.290) | 0.761 |
| CVD mortality | SHS (continuous) | NA | 0.973 (0.879 - 1.075) | 0.587 |
|  | SHS-minima | 140 | 1.000 (NA - NA) | Ref. |
|  | SHS-mild | 83 | 1.281 (0.975 - 1.683) | 0.076 |
|  | SHS-moderate | 43 | 1.010 (0.713 - 1.430) | 0.955 |
|  | SHS-severe | 6 | 1.270 (0.554 - 2.911) | 0.572 |
|  | Sleep pattern 1 | 193 | 1.000 (NA - NA) | Ref. |
|  | Sleep pattern 2 | 20 | 1.426 (0.894 - 2.275) | 0.137 |
|  | Sleep pattern 3 | 36 | 1.110 (0.775 - 1.588) | 0.569 |
|  | Sleep pattern 4 | 23 | 1.046 (0.675 - 1.622) | 0.839 |
|  | Abnormal chronotype | 36 | 1.012 (0.709 - 1.445) | 0.946 |
|  | Long or short sleep duration | 114 | 1.225 (0.960 - 1.563) | 0.103 |
|  | Insomnia | 78 | 0.908 (0.696 - 1.185) | 0.478 |
|  | Napping | 30 | 1.038 (0.706 - 1.527) | 0.849 |
|  | Snoring | 115 | 0.939 (0.733 - 1.204) | 0.621 |
|  | Nonrestorative | 35 | 1.228 (0.856 - 1.763) | 0.265 |
|  | Day time sleepiness | 13 | 0.988 (0.563 - 1.735) | 0.967 |
| anemia | SHS (continuous) | NA | 0.937 (0.901 - 0.974) | 0.001 |
|  | SHS-minima | 954 | 1.000 (NA - NA) | Ref. |
|  | SHS-mild | 473 | 1.117 (1.000 - 1.248) | 0.050 |
|  | SHS-moderate | 318 | 1.177 (1.034 - 1.339) | 0.014 |
|  | SHS-severe | 39 | 1.462 (1.058 - 2.022) | 0.022 |
|  | Sleep pattern 1 | 1274 | 1.000 (NA - NA) | Ref. |
|  | Sleep pattern 2 | 112 | 1.129 (0.929 - 1.373) | 0.222 |
|  | Sleep pattern 3 | 241 | 1.061 (0.924 - 1.219) | 0.402 |
|  | Sleep pattern 4 | 157 | 1.317 (1.113 - 1.558) | 0.001 |
|  | Abnormal chronotype | 244 | 1.108 (0.966 - 1.270) | 0.143 |
|  | Long or short sleep duration | 696 | 1.112 (1.010 - 1.224) | 0.031 |
|  | Insomnia | 596 | 1.115 (1.010 - 1.232) | 0.032 |
|  | Napping | 169 | 1.151 (0.979 - 1.352) | 0.088 |
|  | Snoring | 711 | 0.931 (0.844 - 1.027) | 0.155 |
|  | Nonrestorative | 251 | 1.230 (1.074 - 1.409) | 0.003 |
|  | Day time sleepiness | 101 | 1.343 (1.097 - 1.645) | 0.004 |
| CVD | SHS (continuous) | NA | 0.939 (0.911 - 0.967) | <0.001 |
|  | SHS-minima | 1722 | 1.000 (NA - NA) | Ref. |
|  | SHS-mild | 841 | 1.098 (1.010 - 1.193) | 0.028 |
|  | SHS-moderate | 597 | 1.267 (1.153 - 1.393) | <0.001 |
|  | SHS-severe | 53 | 1.164 (0.884 - 1.534) | 0.279 |
|  | Sleep pattern 1 | 2297 | 1.000 (NA - NA) | Ref. |
|  | Sleep pattern 2 | 193 | 1.185 (1.021 - 1.374) | 0.025 |
|  | Sleep pattern 3 | 469 | 1.245 (1.126 - 1.375) | <0.001 |
|  | Sleep pattern 4 | 254 | 1.176 (1.031 - 1.340) | 0.016 |
|  | Abnormal chronotype | 400 | 1.008 (0.906 - 1.120) | 0.887 |
|  | Long or short sleep duration | 1247 | 1.137 (1.059 - 1.222) | <0.001 |
|  | Insomnia | 1046 | 1.121 (1.041 - 1.208) | 0.003 |
|  | Napping | 297 | 1.062 (0.940 - 1.199) | 0.333 |
|  | Snoring | 1381 | 0.991 (0.922 - 1.066) | 0.814 |
|  | Nonrestorative | 410 | 1.226 (1.103 - 1.362) | <0.001 |
|  | Day time sleepiness | 158 | 1.190 (1.013 - 1.399) | 0.034 |
| Cognitive impairment | SHS (continuous) | NA | 1.005 (0.945 - 1.070) | 0.867 |
|  | SHS-minima | 467 | 1.000 (NA - NA) | Ref. |
|  | SHS-mild | 174 | 0.854 (0.716 - 1.017) | 0.077 |
|  | SHS-moderate | 119 | 0.981 (0.800 - 1.204) | 0.855 |
|  | SHS-severe | 13 | 1.181 (0.677 - 2.061) | 0.558 |
|  | Sleep pattern 1 | 589 | 1.000 (NA - NA) | Ref. |
|  | Sleep pattern 2 | 40 | 1.025 (0.742 - 1.417) | 0.879 |

Table S12. Sensitivity analysis after further adjustment of depression and anxiety

| Outcome | Exposure | Events | HR (95%CI) | P value |
| --- | --- | --- | --- | --- |
| All -cause mortality | SHS (continuous) | NA | 0.979 (0.946 - 1.013) | 0.226 |
|  | SHS-minima | 1299 | 1.000 (NA - NA) | Ref. |
|  | SHS-mild | 663 | 1.112 (1.012 - 1.222) | 0.027 |
|  | SHS-moderate | 406 | 1.043 (0.931 - 1.169) | 0.469 |
|  | SHS-severe | 48 | 1.241 (0.926 - 1.663) | 0.148 |
|  | Sleep pattern 1 | 1749 | 1.000 (NA - NA) | Ref. |
|  | Sleep pattern 2 | 156 | 1.172 (0.993 - 1.384) | 0.061 |
|  | Sleep pattern 3 | 320 | 1.040 (0.923 - 1.173) | 0.519 |
|  | Sleep pattern 4 | 191 | 1.010 (0.868 - 1.176) | 0.896 |
|  | Abnormal chronotype | 316 | 1.005 (0.891 - 1.133) | 0.933 |
|  | Long or short sleep duration | 940 | 1.099 (1.012 - 1.193) | 0.026 |
|  | Insomnia | 777 | 1.057 (0.970 - 1.153) | 0.206 |
|  | Napping | 245 | 1.028 (0.898 - 1.176) | 0.689 |
|  | Snoring | 966 | 0.890 (0.818 - 0.968) | 0.007 |
|  | Nonrestorative | 309 | 1.167 (1.033 - 1.319) | 0.013 |
|  | Day time sleepiness | 117 | 1.063 (0.882 - 1.283) | 0.520 |
| CVD mortality | SHS (continuous) | NA | 0.973 (0.892 - 1.061) | 0.536 |
|  | SHS-minima | 182 | 1.000 (NA - NA) | Ref. |
|  | SHS-mild | 110 | 1.258 (0.991 - 1.597) | 0.059 |
|  | SHS-moderate | 63 | 1.052 (0.784 - 1.410) | 0.736 |
|  | SHS-severe | 7 | 1.036 (0.480 - 2.232) | 0.929 |
|  | Sleep pattern 1 | 256 | 1.000 (NA - NA) | Ref. |
|  | Sleep pattern 2 | 25 | 1.246 (0.821 - 1.892) | 0.301 |
|  | Sleep pattern 3 | 48 | 1.075 (0.788 - 1.467) | 0.648 |
|  | Sleep pattern 4 | 33 | 1.028 (0.710 - 1.487) | 0.885 |
|  | Abnormal chronotype | 50 | 1.039 (0.767 - 1.407) | 0.807 |
|  | Long or short sleep duration | 156 | 1.254 (1.016 - 1.548) | 0.035 |
|  | Insomnia | 107 | 0.920 (0.732 - 1.157) | 0.476 |
|  | Napping | 43 | 1.051 (0.759 - 1.454) | 0.765 |
|  | Snoring | 151 | 0.905 (0.730 - 1.123) | 0.364 |
|  | Nonrestorative | 48 | 1.197 (0.877 - 1.634) | 0.256 |
|  | Day time sleepiness | 18 | 0.945 (0.585 - 1.526) | 0.817 |
| Anemia | SHS (continuous) | NA | 0.935 (0.904 - 0.968) | <0.001 |
|  | SHS-minima | 1232 | 1.000 (NA - NA) | Ref. |
|  | SHS-mild | 630 | 1.123 (1.019 - 1.237) | 0.019 |
|  | SHS-moderate | 431 | 1.189 (1.062 - 1.330) | 0.003 |
|  | SHS-severe | 50 | 1.411 (1.059 - 1.881) | 0.019 |
|  | Sleep pattern 1 | 1659 | 1.000 (NA - NA) | Ref. |
|  | Sleep pattern 2 | 154 | 1.166 (0.986 - 1.379) | 0.072 |
|  | Sleep pattern 3 | 325 | 1.082 (0.960 - 1.220) | 0.198 |
|  | Sleep pattern 4 | 205 | 1.245 (1.074 - 1.443) | 0.004 |
|  | Abnormal chronotype | 324 | 1.118 (0.993 - 1.259) | 0.066 |
|  | Long or short sleep duration | 937 | 1.142 (1.050 - 1.242) | 0.002 |
|  | Insomnia | 784 | 1.088 (0.998 - 1.187) | 0.056 |
|  | Napping | 234 | 1.171 (1.020 - 1.345) | 0.025 |
|  | Snoring | 934 | 0.929 (0.853 - 1.012) | 0.094 |
|  | Nonrestorative | 340 | 1.227 (1.091 - 1.381) | <0.001 |
|  | Day time sleepiness | 131 | 1.271 (1.063 - 1.518) | 0.008 |
| CVD | SHS (continuous) | NA | 0.943 (0.918 - 0.967) | <0.001 |
|  | SHS-minima | 2151 | 1.000 (NA - NA) | Ref. |
|  | SHS-mild | 1092 | 1.109 (1.030 - 1.193) | 0.006 |
|  | SHS-moderate | 775 | 1.248 (1.148 - 1.358) | < 0.001 |
|  | SHS-severe | 69 | 1.102 (0.865 - 1.405) | 0.430 |
|  | Sleep pattern 1 | 2898 | 1.000 (NA - NA) | Ref. |
|  | Sleep pattern 2 | 247 | 1.120 (0.982 - 1.278) | 0.090 |
|  | Sleep pattern 3 | 603 | 1.227 (1.123 - 1.341) | < 0.001 |
|  | Sleep pattern 4 | 339 | 1.176 (1.049 - 1.318) | 0.005 |
|  | Abnormal chronotype | 521 | 1.011 (0.921 - 1.110) | 0.816 |
|  | Long or short sleep duration | 1606 | 1.135 (1.065 - 1.209) | <0.001 |
|  | Insomnia | 1352 | 1.116 (1.045 - 1.193) | 0.001 |
|  | Napping | 393 | 1.051 (0.946 - 1.168) | 0.355 |
|  | Snoring | 1766 | 0.990 (0.929 - 1.055) | 0.760 |
|  | Nonrestorative | 531 | 1.179 (1.074 - 1.295) | <0.001 |
|  | Day time sleepiness | 210 | 1.190 (1.035 - 1.370) | 0.015 |
| Cognitive impairment | SHS (continuous) | NA | 0.999 (0.944 - 1.056) | 0.965 |
|  | SHS-minima | 551 | 1.000 (NA - NA) | Ref. |
|  | SHS-mild | 214 | 0.861 (0.734 - 1.009) | 0.065 |
|  | SHS-moderate | 151 | 1.056 (0.883 - 1.264) | 0.550 |
|  | SHS-severe | 14 | 0.971 (0.568 - 1.661) | 0.914 |
|  | Sleep pattern 1 | 703 | 1.000 (NA - NA) | Ref. |
|  | Sleep pattern 2 | 48 | 0.963 (0.717 - 1.295) | 0.804 |
|  | Sleep pattern 3 | 114 | 0.908 (0.744 - 1.109) | 0.345 |
|  | Sleep pattern 4 | 75 | 1.029 (0.809 - 1.310) | 0.815 |
|  | Abnormal chronotype | 114 | 0.996 (0.817 - 1.215) | 0.971 |
|  | Long or short sleep duration | 345 | 1.007 (0.881 - 1.152) | 0.918 |
|  | Insomnia | 286 | 0.949 (0.825 - 1.092) | 0.465 |
|  | Napping | 94 | 1.044 (0.840 - 1.297) | 0.699 |
|  | Snoring | 354 | 0.915 (0.798 - 1.049) | 0.201 |
|  | Nonrestorative | 113 | 1.140 (0.933 - 1.393) | 0.201 |
|  | Day time sleepiness | 54 | 1.372 (1.040 - 1.810) | 0.025 |
| Hyperparathyroidism | SHS (continuous) | NA | 0.904 (0.823 - 0.992) | 0.034 |
|  | SHS-minima | 171 | 1.000 (NA - NA) | Ref. |
|  | SHS-mild | 70 | 0.982 (0.742 - 1.301) | 0.901 |
|  | SHS-moderate | 69 | 1.602 (1.203 - 2.135) | 0.001 |
|  | SHS-severe | 4 | 1.079 (0.397 - 2.935) | 0.881 |
|  | Sleep pattern 1 | 216 | 1.000 (NA - NA) | Ref. |
|  | Sleep pattern 2 | 24 | 1.438 (0.937 - 2.205) | 0.096 |
|  | Sleep pattern 3 | 47 | 1.159 (0.843 - 1.593) | 0.363 |
|  | Sleep pattern 4 | 27 | 1.547 (1.030 - 2.322) | 0.035 |
|  | Abnormal chronotype | 42 | 1.158 (0.833 - 1.610) | 0.382 |
|  | Long or short sleep duration | 121 | 1.125 (0.894 - 1.415) | 0.316 |
|  | Insomnia | 110 | 1.158 (0.916 - 1.464) | 0.221 |
|  | Napping | 26 | 1.246 (0.828 - 1.875) | 0.293 |
|  | Snoring | 103 | 0.836 (0.656 - 1.067) | 0.150 |
|  | Nonrestorative | 60 | 1.700 (1.274 - 2.270) | <0.001 |
|  | Day time sleepiness | 18 | 1.512 (0.935 - 2.446) | 0.092 |
